# H/D Isotope Effects on ^1^H-NMR Chemical Shifts in Cyclic Heterodimers and Heterotrimers of Phosphinic and Phosphoric Acids

**DOI:** 10.3390/molecules25081907

**Published:** 2020-04-20

**Authors:** Valeriia V. Mulloyarova, Daria O. Ustimchuk, Aleksander Filarowski, Peter M. Tolstoy

**Affiliations:** 1 Institute of Chemistry, St. Petersburg State University, Universitetskij pr. 26, 198504 St. Petersburg, Russia; myllerka20071993@gmail.com (V.V.M.); ustimchuk.d@yandex.ru (D.O.U.); 2 Faculty of Chemistry, University of Wrocław, 14 F. Joliot-Curie str., 50-383 Wrocław, Poland; aleksander.filarowski@chem.uni.wroc.pl

**Keywords:** phosphinic acids, phosphoric acids, hydrogen bond, heterodimers, heterotrimers, heterotetramers, H/D isotope effects, NMR

## Abstract

Hydrogen-bonded heterocomplexes formed by POOH-containing acids (diphenylphosphoric **1**, dimethylphosphoric **2**, diphenylphosphinic **3**, and dimethylphosphinic **4**) are studied by the low-temperature (100 K) ^1^H-NMR and ^31^P-NMR using liquefied gases CDF_3_/CDF_2_Cl as a solvent. Formation of cyclic dimers and cyclic trimers consisting of molecules of two different acids is confirmed by the analysis of vicinal H/D isotope effects (changes in the bridging proton chemical shift, δH, after the deuteration of a neighboring H-bond). Acids **1** and **4** (or **1** and **3**) form heterotrimers with very strong (short) H-bonds (δH ca. 17 ppm). While in the case of all heterotrimers the H-bonds are cyclically arranged head-to-tail, ···O=P–O–H···O=P–O–H···, and thus their cooperative coupling is expected, the signs of vicinal H/D isotope effects indicate an effective anticooperativity, presumably due to steric factors: when one of the H-bonds is elongated upon deuteration, the structure of the heterotrimer adjusts by shortening the neighboring hydrogen bonds. We also demonstrate the formation of cyclic tetramers: in the case of acids **1** and **4** the structure has alternating molecules of **1** and **4** in the cycle, while in case of acids **1** and **3** the cycle has two molecules of **1** followed by two molecules of **3**.

## 1. Introduction

The compounds that have both proton-donating and proton-accepting groups are prone to self-association via hydrogen bonds, depending on the spatial orientation of interacting groups and various steric factors. Perhaps the most classical and well-studied example of such complexation is provided by carboxylic acids, which form cyclic dimers in the gas phase [1], in dilute aprotic solutions [2] and in crystals [3,4], except for formic [5] and acetic [6] acids that crystallize as infinite chains. A somewhat different situation is observed for POOH-containing acids, namely, phosphinic (R_2_POOH) and phosphoric acids ((RO)_2_POOH), which also crystallize as cyclic dimers [7] or infinite chains [8] and form cyclic dimers in the gas phase [9,10,11,12,13], though in some polar aprotic solutions the dominant self-associates are cyclic trimers [14,15]. In our previous works the stoichiometry of the phosphinic and phosphoric acid cyclic trimers has been established by the use of H/D isotope effects on ^1^H-NMR chemical shifts: three hydrogen bonds are mutually coupled and deuteration in one of them changes the geometry and, thus, the NMR parameters of the remaining ones. This phenomenon has been previously called “vicinal H/D isotope effects” [16]. The number of such H/D isotope effects allows one to enumerate the coupled hydrogen bonds in a complex; the signs of the H/D isotope effects enable one to distinguish between cooperative and anticooperative coupling schemes [17]. It has been demonstrated that in the cyclic trimeric self-associates of the phosphinic and phosphoric acids hydrogen bonds are cooperative (mutually strengthening each other), which one would expect for the head-to-tail linking motif [14,15]. While the fact of trimerization is well grounded, the reasons for it are less clear. It has been speculated that the trimers are formed because the OPOH group is not planar and the lone pairs of the P=O group lie out of the OPO plane, thus making the formation of a planar dimer less preferable. In turn, the formation of a cyclic trimer allows molecules to better align their proton-donating and proton-accepting groups to form a non-planar ring of three almost linear hydrogen bonds, in which each OH group points along one of the P=O lone pairs [18]. The non-planarity of the ring of three hydrogen bonds leads to various possible puckering motions in the complex. We also note that the cyclic trimerization of small molecules via hydrogen bonds is rather a rare phenomenon in nature; only pyrazoles [19], 1-amino-3-iminopropenes [20], and possible imidazoles [21] can be mentioned as examples (again, in these cases the structures are governed by the relative orientations of proton-donating NH groups and lone pairs on proton-accepting N atoms). Another distinctive feature of the phosphinic and phosphoric acids self-associates is the unusually high stability: in the gas phase the energies up to 60 kcal/mol per dimethylphosphinic acid self-associate (presumably, cyclic dimer) have been reported previously [9,22]. The complexation constants of phosphinic acids, which are an order of magnitude higher than those typical for carboxylic acids, have been reported [23]. The high stability might be linked to the following: (i) phosphinic acids are reasonably strong proton donors (the p*K*_a_ value is 3.08 for Me_2_POOH [24], 2.32 for Ph_2_POOH [25], 1.85 for (PhO)_2_POOH [25], and 1.25 for (MeO)_2_POOH [25]); (ii) the semipolar P=O group also makes phosphinic acids reasonably strong proton acceptors; and (iii) the internal mobility within a self-associate reduces the entropic penalty upon complexation, which is also another possible reason for the trimerization in solution.

The formation of cyclic heteroassociates, i.e., complexes involving molecules of different acids has been poorly studied. There are a number of publications on the topic of heterodimers of carboxylic acids, studied both experimentally and computationally [26,27,28]. However, we were unable to find any information about the heteroassociates of POOH-containing acids (with one possible exception of the reported mixed carboxylic-phosphoric acid dimer [29]). Therefore, driven by the fundamental interest and curiosity, in this work we investigate the structural and spectroscopic features of hydrogen-bonded complexes that are formed in polar aprotic solution containing two POOH-containing acids.

**Figure 1:**
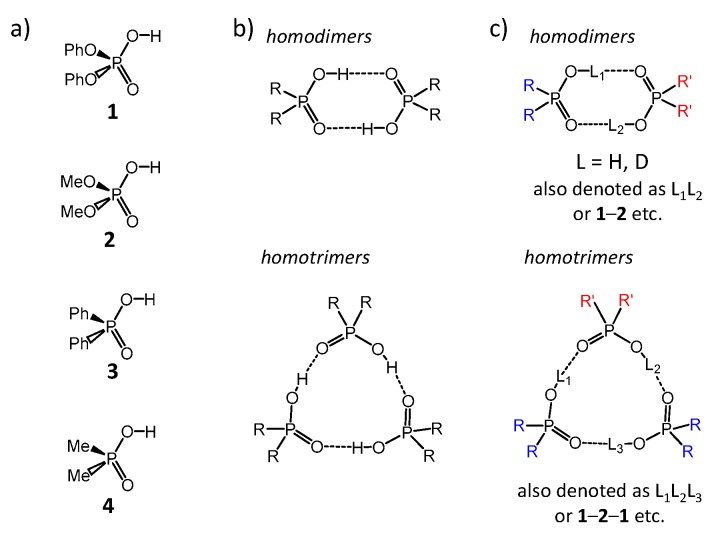
Schematic structures of molecules and their complexes, studied in this work. (**a**) diphenylphosphoric **1**, dimethylphosphoric **2**, diphenylphosphinic **3**, and dimethylphosphinic **4** acids. (**b**) Self-associates of phosphinic and phosphoric acids. (**c**) Heteroassociates of phosphinic and phosphoric acids.

**Figure 2:**
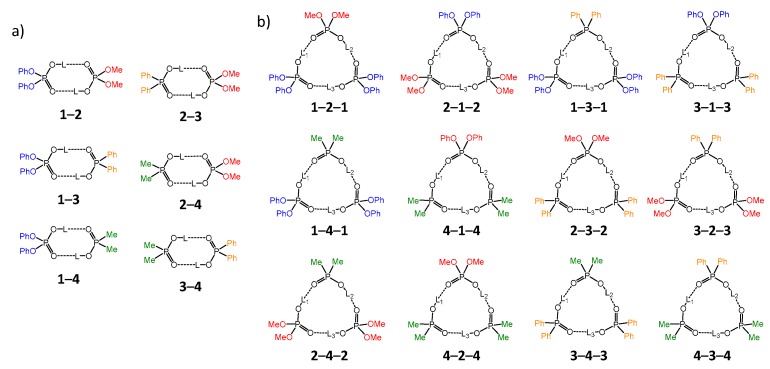
Cyclic heterodimers (**a**) and heterotrimers (**b**) of acids **1**–**4**, studied in this work.

The main molecular objects are shown in Figure 1a: the diphenylphosphoric **1**, dimethylphosphoric **2**, diphenylphosphinic **3**, and dimethylphosphinic **4** acids. The binary mixtures of these acids were investigated by the low-temperature ^1^H-NMR and ^31^P-NMR spectroscopy (down to 100 K) in solution in the liquefied deuterated freonic gases (CDClF_2_/CDF_3_). Under these conditions the proton and molecular exchange processes slowdown in the NMR time scale, resulting in the observation of resolved signals from complexes of different stoichiometry. Previously it has been shown that in freonic solutions acids **1**–**4** self-associate into cyclic dimers and cyclic trimers (Figure 1b) [14,15]. Meanwhile, the stoichiometry, structure, and properties of the mixed complexes—heterodimers and heterotrimers (Figure 1c)—are unknown. Here we report the results of our study of heteroassociates with the help of H/D isotopic effects on ^1^H chemical shifts of bridging protons in the partially deuterated samples (see L = H, D in Figure 1c). Figure 2 shows the schematic structures of all possible cyclic heterodimers (Figure 2a) and cyclic heterotrimers (Figure 2b) of acids **1**–**4** along with the nomenclature referring to these complexes for example heterotrimer, which has acids **1**, **1,** and **2** in the cycle, is denoted as **1**-**2**-**1,** etc. For visual clarity the varying substituents attached to the POOH groups are color-coded. For self-associates we use nomenclature such as **1**-**1** for homodimers and **1**-**1**-**1** for homotrimers. Within the framework of this study the main question is how the heterocomplexation affects (a) hydrogen bond geometries and (b) hydrogen bond mutual coupling.

## 2. Results

### 2.1. Non-Deuterated Complexes

In this section we considered the low-temperature ^1^H-NMR and ^31^P-NMR spectra of samples containing non-deuterated species dissolved in CDF_3_/CDF_2_Cl. The NMR spectra of mixtures of **1** with **2**, **1** with **3,** and **1** with **4** will be presented and assigned. For brevity, we have analyzed step-by-step only the spectra of the mixture of **1** with **2**. The ^1^H-NMR and ^31^P-NMR spectra of combinations of acids **2** with **3**, **2** with **4,** and **3** with **4** are presented in Supporting Information (Figures S1–S6).

**Figure 3:**
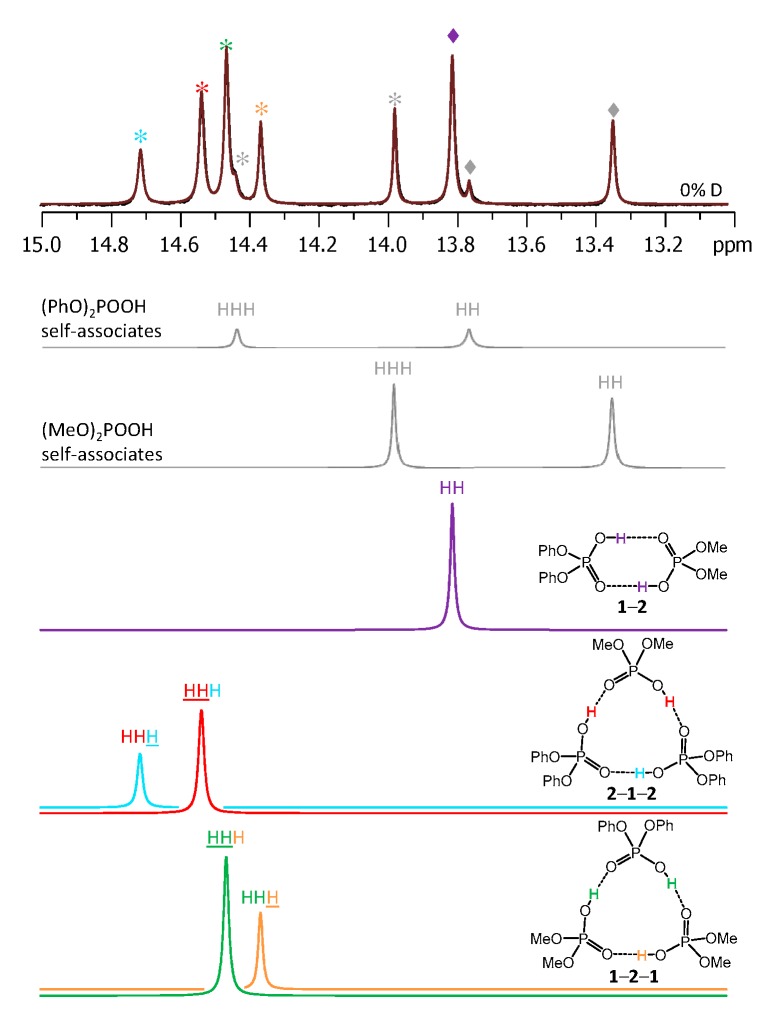
The low-field part of the ^1^H-NMR spectrum of the sample containing acids **1** and **2** (1:1) in CDF_3_/CDF_2_Cl at 100 K. The experimental spectrum is deconvoluted into the sub-spectra arising from self-associated of **1** or **2**, heterodimer **1**-**2,** and two heterotrimers, **1**-**2**-**1** and **2**-**1**-**2**. For visual clarification the signals in the experimental spectrum and the computed sub-spectra are color coded. Trimers and dimers are marked by asterisks and diamonds, respectively.

Figure 3 shows the low-field part of ^1^H-NMR spectrum measured at 100 K of the sample containing diphenylphosphoric acid **1** and dimethylphosphoric acid **2** in the ratio 1:1. In total, nine signals of bridging OH protons are visible in the 13–15 ppm region. Four of these signals were previously assigned to self-associates of acids **1** [15] and **2** [14]: cyclic dimers (marked with grey diamond symbols) and cyclic trimers (grey asterisks). These signals could be subtracted from the spectrum prior to its further deconvolution; the example of such deconvolution is shown in Figure 3 below the experimental spectrum. The experimental spectrum (black curve) and the trace of the sum of all deconvoluted signals (brown curve) match quite well. The remaining five low-field signals belong to heterodimer **1**-**2** and two heterotrimers, **1**-**2**-**1** and **2**-**1**-**2** (the structures are added to Figure 3). The **1**-**2** heterodimer gives rise to one bridging proton signal from two equivalent hydrogen bonds (they become equivalent due to the fast double proton transfer).

**Figure 4:**
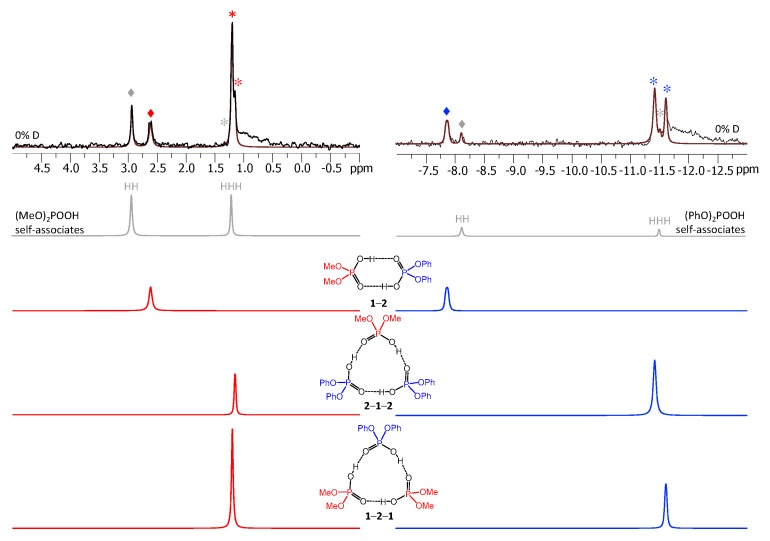
The parts of ^31^P-NMR spectrum of the sample containing acids **1** and **2** (1:1) in CDF_3_/CDF_2_Cl at 100 K. The experimental spectrum is deconvoluted into the sub-spectra arising from self-associated of **1** or **2**, heterodimer **1**-**2,** and two heterotrimers, **1**-**2**-**1** and **2**-**1**-**2**. For visual clarify the signals in the experimental spectrum and the computed sub-spectra are color coded. Trimers and dimers are marked by asterisks and diamonds, respectively.

**Figure 5:**
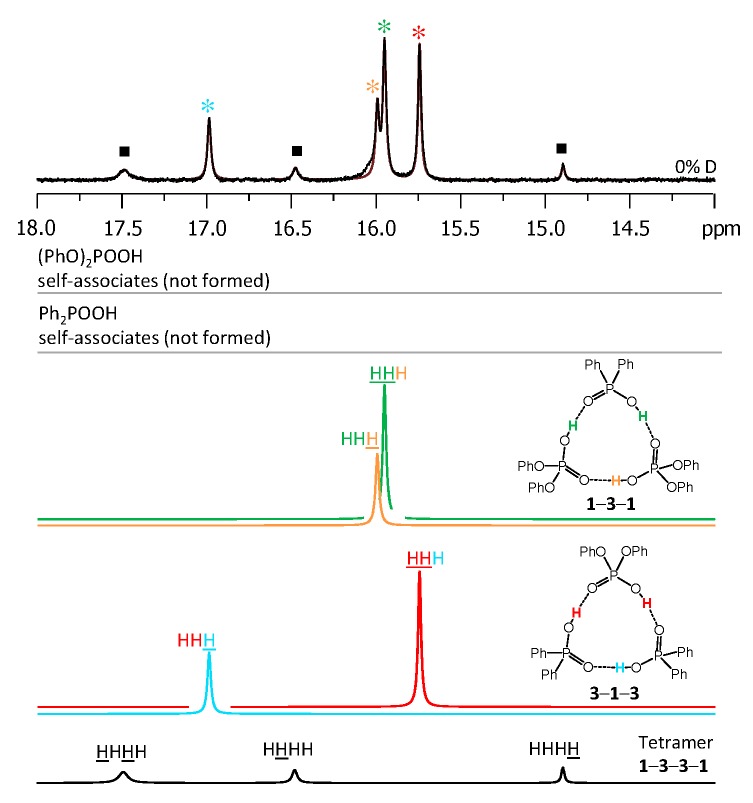
The low-field part of ^1^H-NMR spectrum of the sample containing acids **1** and **3** (1.2:1) in CDF_3_/CDF_2_Cl at 100 K. The experimental spectrum is deconvoluted into the sub-spectra arising from self-associated of **1** or **3**, heterodimer **1**-**3**, two heterotrimers, **1**-**3**-**1** and **3**-**1**-**3**, and a tetramer, consisting of two molecules of **1** and two molecules of **3** in a cyclic **1**-**3**-**3**-**1** configuration. For visual clarification the signals in the experimental spectrum and the computed sub-spectra are color coded. Trimers and tetramers are marked by asterisks and squares, respectively.

**Figure 6:**
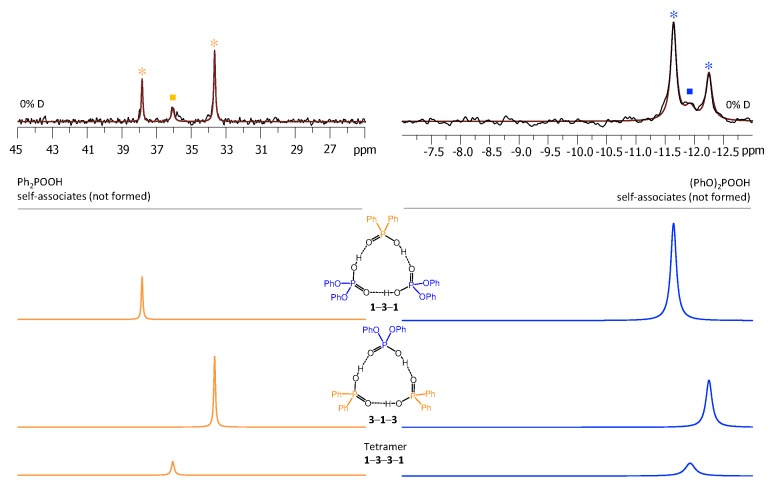
The parts of ^31^P NMR spectrum of the sample containing acids **1** and **3** (1.2:1) in CDF_3_/CDF_2_Cl at 100 K. The experimental spectrum is deconvoluted into the sub-spectra arising from self-associated of **1** or **3**, heterodimer **1**-**3**, two heterotrimers, **1**-**3**-**1** and **3**-**1**-**3**, and a tetramer, consisting of two molecules of **1** and two molecules of **3** in a cyclic **1**-**3**-**3**-**1** configuration. For visual clarification the signals in the experimental spectrum and the computed sub-spectra are color coded. Trimers and tetramers are marked by asterisks and squares, respectively.

**Figure 7:**
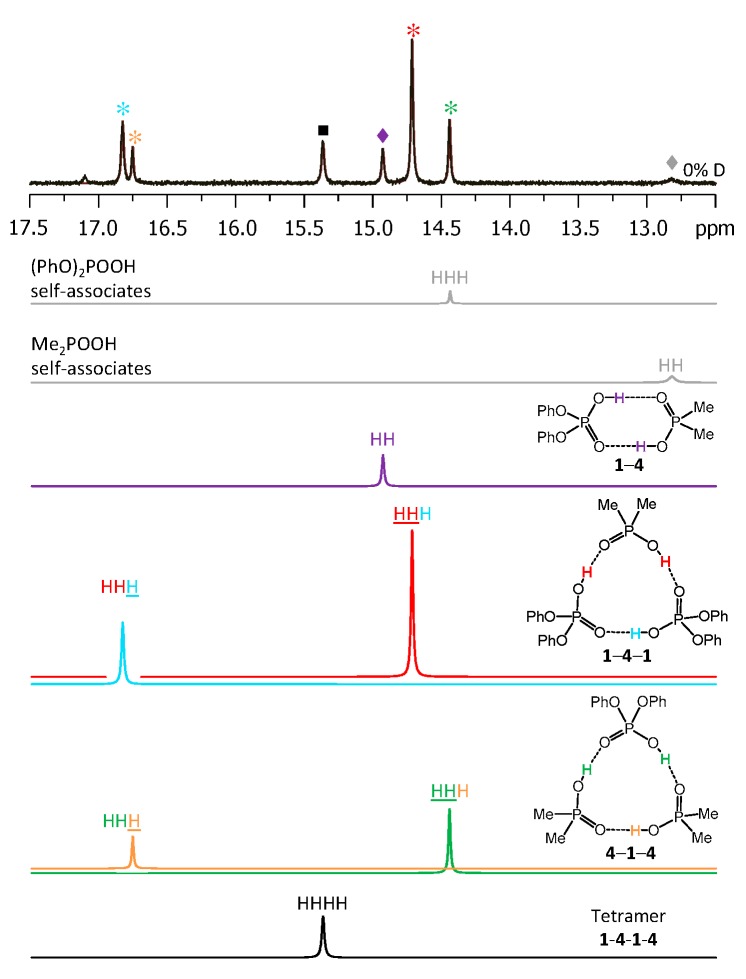
The low-field part of the ^1^H NMR spectrum of the sample containing acids **1** and **4** (1.2:1) in CDF_3_/CDF_2_Cl at 100 K. The experimental spectrum is deconvoluted into the sub-spectra arising from self-associated of **1** or **4**, heterodimer **1**-**4**, two heterotrimers, **1**-**4**-**1** and **4**-**1**-**4,** and a tetramer, consisting of two molecules of **1** and two molecules of **4** in an alternating fashion **1**-**4**-**1**-**4**. For visual clarification the signals in the experimental spectrum and the computed sub-spectra are color coded. Trimers, dimers, and tetramers are marked by asterisks, diamonds, and squares, respectively.

**Figure 8:**
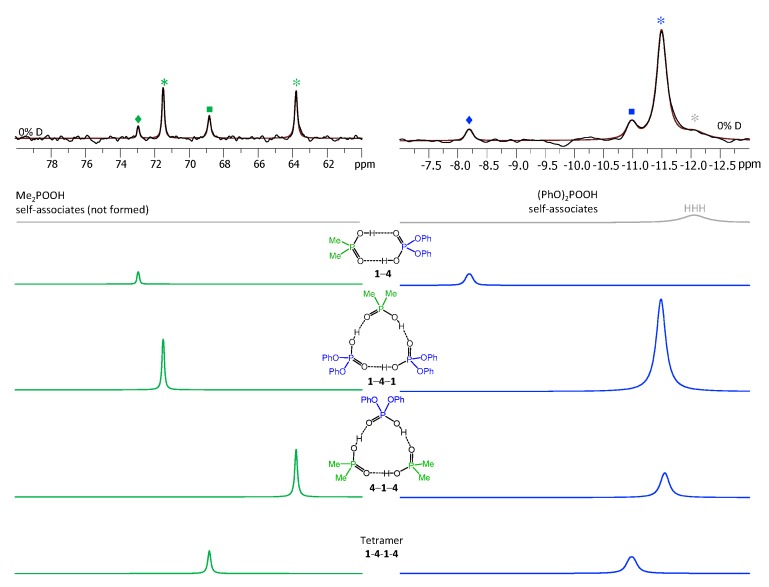
The parts of ^31^P NMR spectrum of the sample containing acids **1** and **4** (1.2:1) in CDF_3_/CDF_2_Cl at 100 K. The experimental spectrum is deconvoluted into the sub-spectra arising from self-associated of **1** or **4**, heterodimer **1**-**4**, two heterotrimers, **1**-**4**-**1** and **4**-**1**-**4,** and a tetramer, consisting of two molecules of **1** and two molecules of **4** in an alternating fashion **1**-**4**-**1**-**4**. For visual clarification the signals in the experimental spectrum and the computed sub-spectra are color coded. Trimers, dimers, and tetramers are marked by asterisks, diamonds, and squares, respectively.

Each heterotrimer gives rise to a pair of signals with relative integrated intensities 2:1. For the heterotrimer **1**-**2**-**1** the more intensive of the signals comes from two protons linking molecules of different acids (**1** and **2**), which indicates the fast triple proton transfer process in the heterotrimer. The less intensive signal comes from one proton linking two molecules of **1**. For the **2**-**1**-**2** heterotrimer the intensities of the signals are reversed. The concentrations of the **1**-**2**, **1**-**2**-**1,** and **2**-**1**-**2** complexes in the sample and the corresponding relative integrated intensities of their signals are not the same, which facilitates assignment. These considerations are almost sufficient for the complete assignment of the spectrum: two pairs of signals with a 2:1 ratio are clearly identifiable, thus the remaining singlet (the most intensive signal in Figure 3) belongs to the **1**-**2** complex. The question remains which pair of signals belongs to **1**-**2**-**1** and which one to **2**-**1**-**2**. The answer could be found in the ^31^P-NMR spectra, shown in Figure 4. Here, after the subtraction of the signals of self-associates (grey symbols), six signals remain: three in the region characteristic for **1** (Figure 4, right; blue symbols) and three signals in the region characteristic for **2** (Figure 4, left; red symbols). Next to the most intensive signals there is also a “hump” stretching to lower chemical shift values; this temperature-dependent feature (visible in a rather narrow temperature range) has been previously observed for self-associates of the phosphinic and phosphoric acids and it was attributed to the slowing of the internal dynamics within the fluxional cycle of hydrogen bonds [15]. Coming back to the assignment at hand, the overall ^31^P-NMR spectrum could be deconvoluted as shown in the bottom part of Figure 4 and the resulting relative integrated intensities of the signals match those in ^1^H-NMR spectrum. Thus, it is possible to assign signals of **1**-**2**-**1** and **2**-**1**-**2** in ^1^H-NMR spectrum: from the ^31^P-NMR spectrum it follows that the concentration of **2**-**1**-**2** is slightly higher than that of **1**-**2**-**1**, i.e., the more intensive pair of signals in ^1^H-NMR spectrum belongs to **2**-**1**-**2**. The complete set of ^1^H-NMR and ^31^P-NMR chemical shifts for **1**-**2**, **1**-**2**-**1,** and **2**-**1**-**2** is listed in Table 1 and Table 2. Similarly, one can assign signals in the ^1^H-NMR and ^31^P-NMR spectra of the samples containing other combinations of acids. The ^1^H-NMR and ^31^P-NMR spectra for a mixture of **1** with **3** are shown in Figure 5 and Figure 6, respectively and for a mixture of **1** with **4** in Figure 7 and Figure 8, respectively. The ^1^H-NMR chemical shifts of all non-deuterated species are collected in Table 1 and Table 2. Three ^1^H-NMR signals in Figure 5 and one of the signals in Figure 7 are marked by a black square; these signals belong neither to self-associates of acids, nor to their heterodimers or heterotrimers. In the next section and in the Discussion we argued that these signals most probably belong to heterotetramers: a cyclic structure of type **1**-**3**-**3**-**1** (two molecules of **1** and then two molecules of **3** in the cycle) in Figure 5 and a cyclic structure of type **1**-**4**-**1**-**4** (alternating molecules of **1** and **4**) in Figure 7.

### 2.2. Partially Deuterated Complexes

The number of signals in the ^1^H-NMR spectra increased upon deuteration. Deuteration in one of the hydrogen bonds causes its geometry to change due to zero-point vibration of the proton/deuteron in the anharmonic bridging particle stretching potential. The geometric H/D isotope effect leads to the changes of the electronic structures of proton-donating and proton-accepting moieties and as a result the effect propagates further to a neighboring hydrogen bond, causing its geometry to change as well. The geometric changes manifest themselves as the change in its ^1^H-NMR chemical shift, a phenomenon that previously was called “vicinal H/D isotope effect” [16]. After the partial deuteration of the sample the deuterons are distributed among all hydroxyl groups, which results in the coexistence of a number of isotopologs. At low temperature the proton/deuteron exchange processes are slowed down in the NMR time scale and the signals of all chemically non-equivalent isotopologs are resolved. The number of new signals appearing for a given complex after its partial deuteration is indicative of the number of mutually interacting hydrogen bonds. For example, a cyclic dimer would have isotopologs HH (two equivalent protons), HD (one proton but a double-degenerated form), and DD; the first two isotopologs would contribute to the ^1^H-NMR spectrum. The relative intensities of the signals of isotopologs are determined mostly by the statistics (i.e., by the random distribution of deuterons) with some corrections due to fractionation factors [30]. The latter means that in most cases the weaker hydrogen bonds are slightly enriched in deuterons and the stronger hydrogen bonds are enriched in protons to the same extent. An example of H/D fractionation and its dependence on the hydrogen bond strength is given in Figure S10 and Table S3 for the mixture of acids **2** and **3** (for this analysis the hydrogen bond strength was assumed to grow monotonously with δH). The observed trend is typical for hydrogen bonded systems, the deuterium enriched weaker hydrogen bonds: for the mixture of acids **2** and **3** the deuteration ratios *x*_D_ of individual complexes fell from 0.67 to 0.54 when the δH values grew from ca. 13 to ca. 15 ppm (see Table S3).

**Figure 9:**
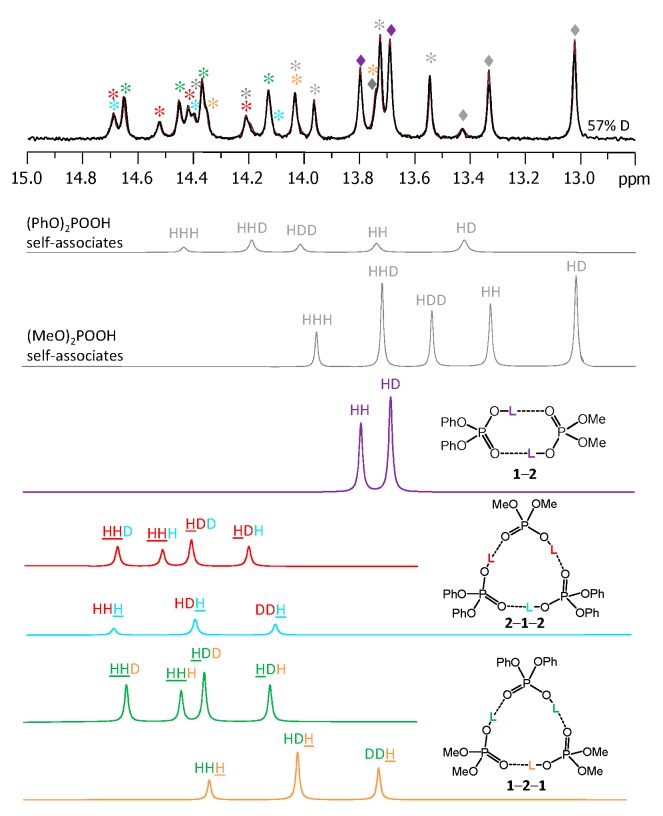
The low-field part of the ^1^H-NMR spectrum of the sample containing partially deuterated (OH/OD, 57% D) acids **1** and **2** (1:1) in solution in CDF_3_/CDF_2_Cl at 100 K. The experimental spectrum is deconvoluted into the sub-spectra arising from various isotopologs of self-associates, heterodimer and heterotrimers. For visual clarification the signals in the experimental spectrum and the computed sub-spectra are color coded. Trimers and dimers are marked by asterisks and diamonds, respectively.

Figure 9 shows the low-field part of the ^1^H-NMR spectrum of the sample containing partially deuterated acids **1** and **2**. Comparing with the spectrum shown in Figure 3, one can see that the number of signals had increased from 9 to 26 (some of the signals overlap, so that the number 26 might not be immediately apparent). Again, using the previously published results [14,15] it is possible to identify signals of self-associates of **1** and **2**: isotopologs HH and HD for cyclic dimers as well as isotopologs HHH, HHD, and HDD for cyclic trimers (in total, this accounted for 10 signals). Here and below we would use the nomenclature of isotopologs as depicted in Figure 1c. Among the remaining sixteen signals five were assigned to non-deuterated complexes **1**-**2**, **1**-**2**-**1,** and **2**-**1**-**2** (i.e., the HH and HHH forms). Thus, only 11 new signals had to be assigned to isotopologs of heterodimers and heterotrimers. One of the signals belonged to the HD isotopolog of **1**-**2**. Two signals come from the HDH and DDH isotopologs of **1**-**2**-**1**; the underlining means that the observed proton was located between molecules of acid **1**, while the deuteration occurred in hydrogen bonds between molecules of acids **1** and **2**. Three more signals come from the HHD, HDH, and HDD isotopologs of **1**-**2**-**1**; here the observed proton was in one of the hydrogen bonds between molecules of acids **1** and **2**, while the deuteration occurred in either one (or both) of the remaining hydrogen bonds. Finally, for the second heterotrimer, **2**-**1**-**2**, there were also five new signals (HDH, DDH, HHD, HDH, and HDD). In this case the ^31^P NMR spectra were not helping the assignment, because within the precision of the measurements the chemical shifts of phosphorous nuclei appeared to be insensitive to deuteration, i.e., the ^31^P-NMR spectra remained virtually unchanged upon deuteration. However, the relative integrated intensities of the ^1^H-NMR signals could be used for assignment: in case of random distribution of deuterons among hydrogen bonds, the proportions between various isotopologs are governed by simple equations as a function of the overall sample deuteration ratio *x*_D_. These equations are given in the Supporting Information below Figure S7. In turn, the *x*_D_ value could be determined from the signal intensities of H/D isotopologs of previously studied homocomplexes or in several cases from the total intensity of the low-field NMR signals in relation to the intensity of the signals of non-exchangeable CH protons. For the spectrum shown in Figure 9 the *x*_D_ value was estimated to be 0.57 (57% D, 43% H). Under these conditions the intensities of the HH and HD signals of heterodimer **1**-**2** were in the 0.43:0.57 ratio. Only one signal fit this ratio and, thus, it could be assigned to the HD isotopolog (signals of **1**-**2** are marked by purple diamonds in Figure 9). Similar logic allowed us to assign signals of heterotrimers as well. Note that the assignment was somewhat facilitated by using the following observations: i) vicinal H/D isotope effects tended to be a few tenths of a ppm in magnitude and also ii) vicinal H/D isotope effects tended to be roughly additive (for example, the shift of the HDD signal from the HHH one is close to the sum of the shifts of the HHD and HDH signals). Using this approach we deconvoluted the experimental spectrum into a series of sub-spectra from the set of isotopologs of each complex (see the bottom part of Figure 9). The sum of sub-spectra matched the experimental spectrum quite well (black and brown traces in Figure 9).

**Figure 10:**
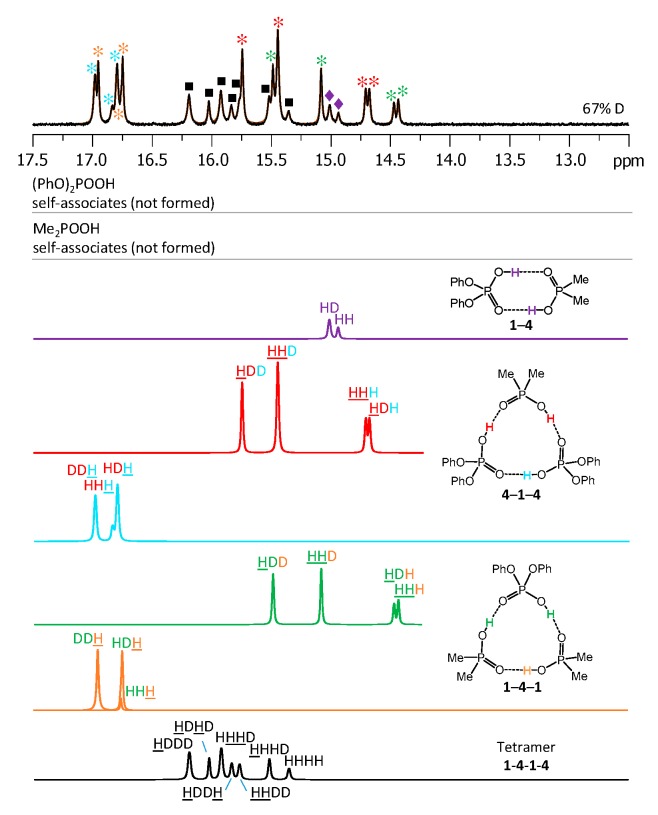
The low-field part of the ^1^H-NMR spectrum of the sample containing partially deuterated (OH/OD, 67% D) acids **1** and **4** (1.2:1) in solution in CDF_3_/CDF_2_Cl at 100 K. The experimental spectrum is deconvoluted into the sub-spectra arising from various isotopologs of self-associates, heterodimer, heterotrimers, and a heterotetramer. For visual clarification the signals in the experimental spectrum and the computed sub-spectra are color coded.

The low-temperature ^1^H-NMR spectrum of samples containing a partially deuterated mixture of acids **1** with **4** is shown in Figure 10. At the very bottom of Figure 10 the deconvolution of the experimental spectrum gives seven extra signals, which are addressed in the Discussion. The ^1^H-NMR spectra of some other mixtures of the partially deuterated acids (**2** with **3** and **3** with **4**) are shown in Supporting Information in Figures S8 and S9. The resulting list of chemical shifts is given in Table 1 and Table 2. We did not manage to obtain the resolved spectra of mixtures of partially deuterated acids **1** with **3** and **2** with **4**, so this information is missing in Table 1 and Table 2.

## 3. Discussion

In this section we focused on heterocomplexes. For the analysis of the signals of homocomplexes see references [14,15], where the spectral patterns for homodimers and homotrimers are presented for the acids studied in this work, as well as for the non-symmetrically substituted phosphinic acid PhHPOOH and the enantiomers of bis(2,4,4-trimethylpentyl)phosphinic acid (in the latter two cases the loss of symmetry of the trimer leads to the coexistence of its chemically non-equivalent isomers and more complicated spectral patterns).

Here we will discuss three specific features that seem to be unique for the heterocomplexes of phosphinic and phosphoric acids: (i) the unusual hydrogen bond strength, (ii) the effective hydrogen bond anticooperativity despite the cyclic structure of heterocomplexes, and (iii) the evidence for the tetramer formation.

### 3.1. Hydrogen Bond Strength

The relative strengths of hydrogen bonds in heterocomplexes of acids **1**–**4** could be assessed on the basis of the absolute values of the ^1^H-NMR chemicals shifts, δH. There is a number of publications where linear correlations between hydrogen bond strength and the shift of the bridging proton signal upon complexation, ΔδH = δH − δH_free_ were proposed [31,32,33,34,35,36]. The coefficients of proportionality differ for various types of complexes but the relations tend to remain linear within a given homologous series. Assuming that the chemical shifts of the hypothetical free OH groups for acids **1**–**4** are roughly the same, we take the δH value as a measure of the hydrogen bond strength: the further the signal is shifted to low-field, the stronger is the corresponding hydrogen bond. It was shown previously that δH values in OHO hydrogen bonds monotonously increase with the shortening of the O...O distance (and concurrent hydrogen bond symmetrization) [37], therefore, we used the terms “elongation” and “weakening” interchangeably. From Table 1 and Table 2 it follows that the δH values of self-associates did not correlate with the p*K*_a_ values of acid molecules (see Introduction for the list of p*K*_a_ values of **1**–**4**). However, there is a tendency within the set: the molecule that formed weaker cyclic homodimers also formed weaker cyclic homotrimers. When it comes to heterodimers, the strongest hydrogen bonds are formed when two interacting molecules belong to different “types”: one is phosphinic acid and the other is phosphoric acid, as in complexes **1**-**3** and **1**-**4**. For heterotrimers there is a variety of cases and it is hard to make out an overall trend. However, the strongest hydrogen bonds in heterotrimers with δH values close to 17 ppm were formed between two molecules of phosphoric acid **1** when the third molecule was a phosphinic acid, either **3** or **4** (see HHH forms of complexes **1**-**3**-**1** and **1**-**4**-**1** in Table 2). In contrast, the smallest values of δH around 14.5 ppm were observed mostly for heterotrimers formed either by phosphoric acids exclusively (**1**-**2**-**1** and **2**-**1**-**2**) or by phosphinic acids exclusively (**3**-**4**-**3** and **4**-**3**-**4**). There were exceptions to these rules, though, so it is still hard to generalize. The δH values for heterocomplexes were larger in average than those for homocomplexes; from this observation one could conclude that the hydrogen bonds were stronger and it would be interesting to check if the equilibrium between homo- and heterocomplexes would be shifted towards the latter in the gas phase as well. To our knowledge, the value of 17 ppm is the largest chemical shift value for an intermolecular hydrogen bond in neutral self-associate of an acid, including the POOH- and COOH-containing ones (an intramolecular bonding [38] or strong steric hindrance [39] might lead to larger values still).

### 3.2. Cooperativity And Anticooperativity

In the crystal structures of phosphinic and phosphoric acids the POOH groups are always linked in such a way that each POOH group serves as a proton donor to the neighboring POOH group and at the same time it serves as a proton acceptor to the same or a different POOH group (depending on whether cyclic dimers or infinite chains are formed in the crystal state). For example, acid **4** crystallizes as an infinite chain [8], while larger substituents in the case of acid **3** lead to the formation of cyclic dimers [7]. The crystal structures of acids **1** and **2** are unknown, though a similar bis(*p*-methylphenyl)-phosphoric acid, (4-Me-PhO)_2_POOH, forms cyclic dimers [40]. In any case, the principal motifs are invariably of the type ⋅⋅⋅O=P–O–H⋅⋅⋅O=P–O–H⋅⋅⋅, either in a chain or in a cycle, which is also true for the homo- or heterodimers and trimers studied in this work. For such head-to-tail configurations one would expect a cooperative coupling of hydrogen bonds [41,42], which means that if one of the bonds elongates, then the neighboring bonds elongate as well, due to the electronic coupling of the proton-donating and proton-accepting moieties within the POOH group. The opposite case—geometric anticooperativity—would be expected if two proton donors compete for the same proton acceptor, as in case of some homoconjugated anions [17]. In our studies the weakening (elongation) of the hydrogen bond was caused by the H/D substitution. The effect of deuteration on the neighboring bonds was monitored by the changes of the ^1^H-NMR chemical shifts of the remaining bridging protons. The hydrogen bond elongation manifested itself as a high-field shift (cooperativity, negative vicinal H/D isotope effect), while the hydrogen bond contraction led to the low-field shift (anticooperativity, positive vicinal H/D isotope effect). All self-associates (homodimers and homotrimers) featured cooperativity, namely, isotopologs HD (for dimers), HHD, and HDD (for trimers) gave rise to the signals in higher fields than the signals of corresponding non-deuterated species. Such cooperativity was reported previously for self-associates of phosphinic and phosphoric acids [14,15] and for carboxylic acids [17,39]. However, the situation was different for some heterocomplexes. For example, the hydrogen bonds in heterodimer **1**-**4** (which includes one phosphinic and one phosphoric acid) were effectively anticooperatively coupled: deuteration of one of the hydrogen bonds led to the low-field shift of the signal of the remaining bridging proton, δ(HD) – δ(HH) > 0 ppm. This means that the electronic coupling within the POO group was dominated by a different effect. Presumably, it might be a geometric feature: the elongation of one hydrogen bond changed the “packing” of acid’s molecules in the heterodimer in such a way that the other bond contracted, showing an effective anticooperativity. Moreover, for each heterotrimer for which the set of H/D isotope effects was measured there was at least one hydrogen bond that was effectively anticooperatively coupled to the other: the signals of some isotopologs were shifted to lower fields, as compared to the non-deuterated form HHH. For example, for heterotrimers **1**-**2**-**1** and **2**-**1**-**2** (Figure 9) the vicinal H/D isotope effect δ(HHD) – δ(HHH) was positive, while the other vicinal H/D isotope effect, δ(HDH) – δ(HHH), was negative. This means that hydrogen bonds linking two molecules of the same acid were cooperative coupled to each other and anticooperatively coupled to the hydrogen bond linking molecules of two different acids.

The phenomenon of effective anticooperativity of hydrogen bonds involving POOH groups in a head-to-tail configuration and the hypothesis of the “packing” origins of this phenomenon, however, require a further theoretical investigation, which was beyond the scope of this paper.

### 3.3. A case for the Tetramer

The spectra shown in Figure 5, Figure 6, Figure 7 and Figure 8 contain NMR signals of hydrogen bonded species that were tentatively assigned to heterotetramers (see the square markers in the Figures). In this subsection we summarized the evidence for the tetramerization and proposed the structures of these tetramers.

**Figure 11:**
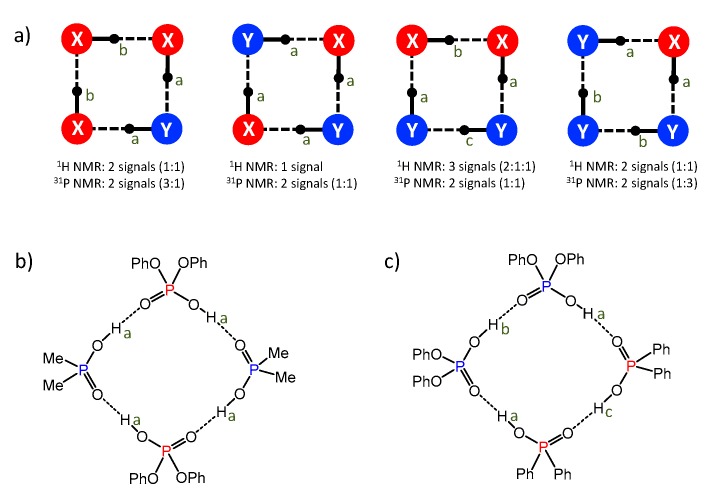
(**a**) The schematic representation of four possible structures of cyclic heterotetramers, formed by two different molecules (blue and red in this case); letters a, b and c indicate chemically non-equivalent bridging protons. (**b**) The proposed structure of heterotetramer of acids **1** and **4** (matches the spectral pattern seen in Figure 7 and Figure 8). (**c**) The proposed structure of heterotetramer of acids **1** and **3** (matches the spectral pattern seen in Figure 5 and Figure 6).

(1) Above we successfully identified signals belonging to homodimers, homotrimers, heterodimers, and two types of heterotrimers, thus the next possible simplest complex involves four acid molecules. Under the experimental conditions used, homotetramers are not formed [14,15], so the signals marked with squares in Figure 5, Figure 6, Figure 7 and Figure 8 belong to heterotetramers. Moreover, these heterotetramers are likely to be cyclic structures, because all OH groups in this complex resonate at low field, i.e., all OH groups are involved in hydrogen bonds of a similar strength.

(2) If we labeled two acids as **X** and **Y**, the four possible cyclic tetramer structures would have the motifs **X**-**Y**-**Y**-**Y**, **X**-**Y**-**X**-**Y**, **Y**-**X**-**X**-**Y,** and **Y**-**X**-**X**-**X** (the ends of these sequences are linked to each other). These motifs have different numbers of non-equivalent bridging protons and phosphorous nuclei, i.e., different numbers of the ^1^H-NMR and ^31^P-NMR signals, as schematically depicted in Figure 11a (in each case we have assumed the average symmetry of the complex, which is achieved after a quadruple proton transfer, fast in the NMR time scale).

In our NMR spectra (see Figure 7 and Figure 8), the heterotetramer of acids **1** and **4** features one ^1^H-NMR signal and two ^31^P-NMR signals of equal intensities (one in the spectral range characteristic for acid **1** and the other characteristic for acid **4**). Thus, acids **1** and **4** form heterotetramer with the alternating pattern **1**-**4**-**1**-**4**, as shown in Figure 11b. The heterotetramer of acids **1** and **3** (Figure 5 and Figure 6) has three ^1^H NMR signals (relative intensities 2:1:1) and two ^31^P-NMR signals with equal intensities. The only structure that fits this spectral pattern is **1**-**3**-**3**-**1**, as shown in Figure 11c.

(3) After the partial deuteration, the heterotetramer **1**-**4**-**1**-**4** gave rise to seven signals in the ^1^H-NMR spectrum (Figure 10): One from the non-deuterated species HHHH (at 15.35 ppm) and six signals from partially deuterated isotopologs (at 15.52, 15.77, 15.83, 15.92, 16.02, and 16.19 ppm). The number of signals matched the expected one: HHHH, HHHD and HHHD, HDHD, HDDH, HHDD, and HDDD. The assignment of the signals could be done on the basis of their relative integrated intensities and assumption of the statistical distribution of deuterons, in a way very much the same as for heterotrimers (see Figure S7 for the more details).

We believed that the combination of the three abovementioned arguments builds a strong case for the formation of heterotetramers of acids **1** and **4** (**1**-**4**-**1**-**4**) as well as acids **1** and **3** (**1**-**3**-**3**-**1**). Such tetramerization, being an even more rare type of complexation of small molecules than trimerization, most likely is governed by the same factors as trimerization: the non-planarity of the ring of hydrogen bonds (the directions of proton-accepting and proton-donating abilities lie out of the POO plane) and the high residual mobility of the tetramer due to various possible puckering motions [18]. Finally, there might be two reasons why homotetramers are not formed, while heterotetramers are: the larger dipole moment of heterotetramers could stabilize them in the polar aprotic medium and the molecules of phosphinic and phosphoric acids might be packing more conveniently in the mixed complex than in a self-associate, due to steric effects.

## 4. Materials and Methods

### 4.1. Sample Preparation

The required weights of acids **1**–**4** were dissolved in deuterated chloroform (CDCl_3_, 99.8% D, Eurisotop) to prepare the 0.01 M solutions. Then equimolar amounts of acids were transferred into a thick-walled NMR sample tube with a J. Young valve (Wilmad 522-LPV-7) and CDCl_3_ was removed in vacuo. After that, the mixture of deuterated freonic gases CDF_3_/CDF_2_Cl, synthesized by a modified method of [43] was added by vacuum transfer (at ca. 10^−6^ mbar). Prior to the addition to the sample the CDF_3_/CDF_2_Cl mixture was additionally dried over alumina in a cold isopropanol bath under vacuum. Due to the lower solubility of phosphinic/phosphoric acids in the CDF_3_/CDF_2_Cl mixture than in CDCl_3_ the resulting ratios of acids in many samples (measured as NMR signal intensities at low temperature) were slightly different than 1:1 (ranging from 1:1.3 to 1:1.8). The absolute concentrations of acids in the samples were 0.004–0.01 M. As diphenylphosphinic acid **3** is poorly soluble both in CDCl_3_ and in CDF_3_/CDF_2_Cl mixture, in some cases the starting solution of **3** in CDCl_3_ was heated to ca. 50 °C to increase the solubility. In one of the samples containing acids **3** and **4** the acids were taken in ratio 1:22 in order to facilitate the spectral assignment by shifting the equilibrium towards the **4**-**3**-**4** heterotrimer (see also Figure S10).

The preparation procedure for partially deuterated samples differed from that for non-deuterated ones in the following: after the transfer of acids to a sample tube and before the addition of the freonic solvent, CH_3_OD (about 20–80 μl; 99.0% D, Eurisotop) was added to the sample tube and subsequently removed in vacuo. The degrees of deuteration were estimated from NMR spectra at low temperature (100 K) based on the relative signal intensities of the remaining OH groups and non-exchangeable CH groups; for individual complexes the degrees of deuteration were determined based on the relative integrated signal intensities of various complex’s isotopologs.

### 4.2. NMR Measurements

The low-temperature (100 ± 1 K) liquid-state ^1^H-NMR and ^31^P-NMR spectra were recorded at the Center of Magnetic Resonance in Saint-Petersburg State University Research Park on Bruker (Germany) Avance III 500 MHz spectrometer (11.74 T, 500.03 MHz for ^1^H, 202.42 MHz for ^31^P). Acquisition parameters for ^1^H: 30°-pulses, acquisition time of 2.6 s, the relaxation delay 6.5 s, 64 scans, no ^31^P decoupling. Acquisition parameters for ^31^P: ^1^H decoupling, 30°-pulses, acquisition time of 0.8 s, the relaxation delay 6.5 s, number of scans ranging from 128 to 512, depending on the sample. No detectable *J*(P,H) couplings were observed with bridging protons, so turning off ^1^H decoupling for ^31^P NMR spectra or turning on ^31^P decoupling for ^1^H-NMR spectra did not affect the appearance of the signals. The ^1^H-NMR spectra were calibrated to TMS scale using the central signal of CHClF_2_ triplet as an internal secondary standard set to 7.21 ppm. The ^31^P-NMR spectra were referenced to H_3_PO_4_ (85% in H_2_O) using the unified Ξ scale, according to IUPAC recommendations [44].

The sample compositions given in the figure’s captions were estimated from the integral intensities of CH proton signals at room temperature. At low temperature sample compositions might change due to different solubilities of acids in the mixture. However, for the samples involving acids **1** and **3** it is difficult to estimate the new composition because at low temperature the aromatic CH signals are significantly broadened and overlap with the solvent peaks.

### 4.3. NMR Spectra Deconvolution

The spectral deconvolution was done by least-square fitting the experimental NMR spectra to a sum of several Lorentzian line shapes. For the spectra of non-deuterated species all the parameters of individual Lorentzians were fitted: position, peak intensity, and linewidth. The positions of the resulting Lorentzians were fixed during the fitting of the spectra of partially deuterated species (the line widths were slightly corrected in some cases). In other words, only the signals of partially deuterated isotopologs were fully fitted. No signal normalization was required during the fitting. The overall set of fitted signals was then split into several groups, each belonging to a particular homo- or heterocomplex. This splitting was based on three features: (i) the previously published vicinal isotope effect values for homocomplexes [14,15]; (ii) the similarity of the line widths within the group for heterocomplexes and (iii) the distribution of integrated intensities within the group that matched both the statistical probabilities of isotopologs and the overall deuteration ratio of the sample (it works for dimer, dimer, and tetramers alike, see Figure S7 for the corresponding statistical equations and plots; in several cases for additional certainty spectra of the samples with different deuteration ratios were compared as well). This procedure usually resulted in a unique way of signal assignment, meaning that alternative assignment patterns had internal contradictions.

## 5. Conclusions

In this work we considered POOH-containing acids **1**–**4**, in particular, the mixed hydrogen bonded complexes formed by molecules of two different acids in the polar aprotic solution. In the majority of cases cyclic heterodimers and cyclic heterotrimers were formed. The stoichiometry of these heterocomplexes was established by counting the number of vicinal H/D isotope effects on the ^1^H-NMR chemical shifts and by analyzing the signal intensities in the ^1^H and ^31^P NMR spectra. It is shown that the heterotrimers containing a mixture of phosphinic and phosphoric acids (as in **1**-**4**-**1** or **1**-**3**-**1**) contain probably the strongest hydrogen bond among the previously reported ones for neutral self-associates: bridging proton chemical shift is ca. 17 ppm. Another interesting feature of heterotrimers is associated with the mutual influence of hydrogen bonds. In all cases hydrogen bonds in the cycle are arranged in a head-to-tail motif, ⋅⋅⋅O=P–O–H⋅⋅⋅O=P–O–H⋅⋅⋅ and thus one could expect a cooperative coupling of these bonds (similar to the case of cyclic dimers of carboxylic acids [17,39] or self-associates of phosphinic and phosphoric acids [14,15]). However, the signs of vicinal H/D isotope effects are evidence of an effective anticooperativity: deuteration (lengthening) of one of the hydrogen bonds leads to the shortening of the neighboring ones. We tentatively attribute this phenomenon to steric factors: apparently when one of the H-bond is elongated upon deuteration, the optimal “packing” of molecules in a non-planar cyclic trimer requires neighboring bonds to slightly contract—an effect that overcomes the expected electronic cooperativity. Finally, the evidence of the formation of cyclic tetramers of the type **1**-**4**-**1**-**4** (alternating molecules of **1** and **4** in a cycle) and **1**-**3**-**3**-**1** (two molecules of **1** followed by two molecules of **3** in a cycle) was presented.

## Figures and Tables

**Table 1:** The ^1^H-NMR chemical shifts of bridging proton in various isotopologs of homodimers and heterodimers of POOH-containing acids **1**–**4** in CDF_3_/CDF_2_Cl at 100 K. The corresponding spectra are shown in Figure 3, Figure 5, Figure 7, Figure 9 and Figure 10 and Figures S1, S3, S5, S8, and S9.

Complex	HH	HD
**1**-**1**^a^	13.75	13.43
**2**-**2**^b^	13.33	13.02
**3**-**3**^c^	n.d. ^d^	n.d.
**4**-**4**^a^	12.75	12.47
**1**-**2**	13.80	13.69
**1**-**3**	n.d.	n.d.
**1**-**4**	14.93	15.00
**2**-**3**	15.10	14.82
**2**-**4**	13.74	n.m. ^e^
**3**-**4**	15.08	n.d.

^a^—chemical shifts coincide with those reported previously in reference [15]. ^b^—chemical shifts coincide with those reported previously in reference [14]. ^c^—the diphenylphosphinic acid **3** is poorly soluble in CDF_3_/CDF_2_Cl and does not form self-associates in a detectable amount. ^d^—n.d.—not detected. ^e^—n.m.—not measured.

**Table 2:** The ^1^H-NMR chemical shifts of bridging protons in various isotopologs of homotrimers and heterotrimers of POOH-containing acids **1**–**4** in CDF_3_/CDF_2_Cl at 100 K. The corresponding spectra are shown in Figure 3, Figure 5, Figure 7, Figure 9 and Figure 10 and Figures S1, S3, S5, S8, and S9.

Complex	HHH	HHD	HDH	HDD	HHH	HDH	DDH
**1**-**1**-**1**^a^	-	-	-	-	14.45	14.28	14.11
**2**-**2**-**2**^b^	-	-	-	-	13.96	13.72	13.54
**3**-**3**-**3**^c^	-	-	-	-	n.d. ^d^	n.d.	n.d.
**4**-**4**-**4**^a^	-	-	-	-	13.76	13.54	13.38
**1**-**2**-**1**	14.53	14.70	14.22	14.43	14.71	14.41	14.12
**2**-**1**-**2**	14.46	14.66	14.14	14.38	14.36	14.04	13.74
**1**-**3**-**1**	15.74	n.m. ^e^	n.m.	n.m.	16.98	n.m.	n.m.
**3**-**1**-**3**	15.94	n.m.	n.m.	n.m.	15.98	n.m.	n.m.
**1**-**4**-**1**	14.70	15.44	14.67	15.75	16.83	16.97	16.78
**4**-**1**-**4**	14.43	15.07	14.46	15.48	16.76	16.94	16.74
**2**-**3**-**2**	14.98	15.02	14.64	14.75	14.29	13.97	13.62
**3**-**2**-**3**	14.72	14.75	14.40	14.48	15.39	n.d.	n.d.
**2**-**4**-**2**	14.80	n.m.	n.m.	n.m.	15.04	n.m.	n.m.
**4**-**2**-**4**	14.81	n.m.	n.m.	n.m.	14.88	n.m.	n.m.
**3**-**4**-**3**	14.50	n.d.	n.d.	n.d.	15.24	n.d.	n.d.
**4**-**3**-**4**	14.61	14.67	14.34	14.44	13.83	13.55	13.30

^a^—chemical shifts coincide with those reported previously in reference [15]. ^b^—chemical shifts coincide with those reported previously in reference [14]. ^c^—the diphenylphosphinic acid **3** is poorly soluble in CDF_3_/CDF_2_Cl and does not form self-associates in a detectable amount. ^d^—n.d.—not detected. ^e^—n.m.—not measured.

## Supplementary Materials

The following are available online at, Figure S1: ^1^H-NMR spectrum of a mixture of acids **2** and **3**, Figure S2: ^31^P-NMR spectrum of a mixture of acids **2** and **3**, Figure S3: ^1^H-NMR spectrum of a mixture of acids **2** and **4**, Figure S4: ^31^P-NMR spectrum of a mixture of acids **2** and **4**, Figure S5: ^1^H-NMR spectrum of a mixture of acids **3** and **4**, Figure S6: ^31^P-NMR spectrum of a mixture of acids **3** and **4**, Figure S7: probabilities of various isotopologs and relative intensities of their signals as a function of deuteration ratio *x*_D_, Figure S8: ^1^H-NMR spectrum of a mixture of partially deuterated acids **2** and **3**, Figure S9: ^1^H-NMR spectra of a mixture of partially deuterated acids **3** and **4** at various degrees of deuteration, Table S1: ^31^P-NMR chemical shifts of homodimers and heterodimers of acids **1**–**4**, Table S2: ^31^P-NMR chemical shifts of homotrimers and heterotrimers of acids **1**–**4**.
