# Supplementary material for: H/D Isotope Effects on 1H-NMR Chemical Shifts in Cyclic Heterodimers and Heterotrimers of Phosphinic and Phosphoric Acids
Source: Molecules. 2020 Apr 20;25(8):1907. doi: 10.3390/molecules25081907 (PMC7221807; doi:10.3390/molecules25081907)
Supplement: Supplementary file 1 [file molecules-25-01907-s001.pdf]

Supporting information for

H/D Isotope Effects on ^{1}H NMR Chemical Shifts in Cyclic Heterodimers and Heterotrimers of Phosphinic and Phosphoric Acids

Valeria V. Mulloyarova[1], Daria O. Ustimchuk[1], Aleksander Filarowski[2], Peter M. Tolstoy[1,*]

[1] Institute of Chemistry, St. Petersburg State University, Universitetskij pr. 26, 198504 St. Petersburg, Russia; myllerka20071993@gmail.com (V.V.M.); ustimchuk.d@yandex.ru (D.O.U.)

[2] Faculty of Chemistry, University of Wrocław, 14 F. Joliot-Curie str., 50-383 Wrocław, Poland; aleksander.filarowski@chem.uni.wroc.pl

* Correspondence: peter.tolstoy@spbu.ru; tel.: +7-921-430-8191

Contents **Page**

Figure S1. The low-field part of ^{1}H NMR spectrum of a mixture of acids **2** and **3**. 2

Figure S2. Parts of ^{31}P NMR spectrum of a mixture of acids **2** and **3**. 3

Figure S3. The low-field part of ^{1}H NMR spectrum of a mixture of acids **2** and **4**. 4

Figure S4. Parts of ^{31}P NMR spectrum of a mixture of acids **2** and **4**. 5

Figure S5. The low-field part of ^{1}H NMR spectrum of a mixture of acids **3** and **4**. 6

Figure S6. Parts of ^{31}P NMR spectrum of a mixture of acids **3** and **4**. 7

Figure S7. Probabilities of various isotopologs and relative intensities of their signals as a function of deuteration ratio x_D. 8

Figure S8. The low-field part of ^{1}H NMR spectrum of a mixture of partially deuterated acids **2** and **3**. 9

Figure S9. The low-field parts of ^{1}H NMR spectra of a mixture of partially deuterated acids **3** and **4** at various degrees of deuteration. 10

Table S1. ^{31}P NMR chemical shifts of homodimers and heterodimers of acids **1**–**4**. 11

Table S2. ^{31}P NMR chemical shifts of homotrimers and heterotrimers of acids **1**–**4**. 12

Figure S10. The analysis of H/D fractionation factors for a mixture of partially deuterated acids **2** and **3**. 13

Table S3. The deuteration ratios of individual hydrogen bonds for the sample of a mixture of partially deuterated acids **2** and **3**. 13

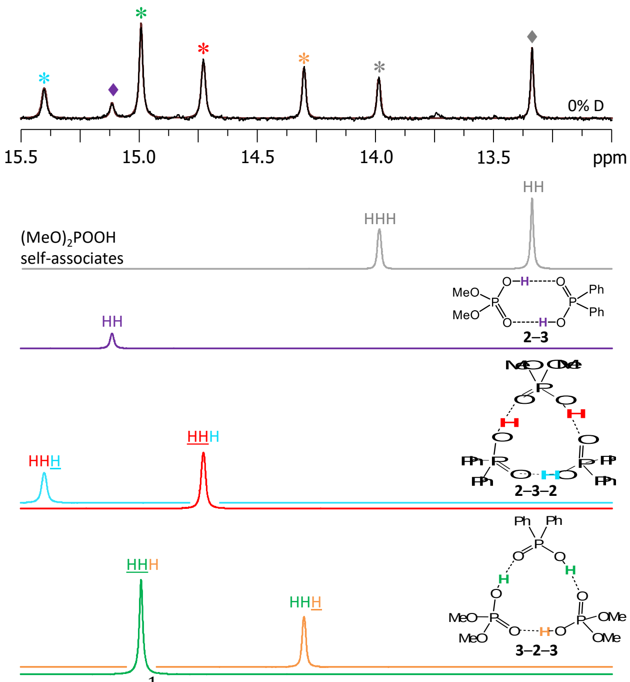

Figure S1. The low-field part of ^{1}H NMR spectrum of the sample containing acids **2** and **3** (1.8:1) in CDF_3/CDF_2Cl at 100 K. The experimental spectrum is deconvoluted into the sub-spectra arising from self-associated of **2**, heterodimer **2-3** and two heterotrimers, **2-3-2** and **3-2-3**. For visual clarify the signals in the experimental spectrum and the computed sub-spectra are color coded.

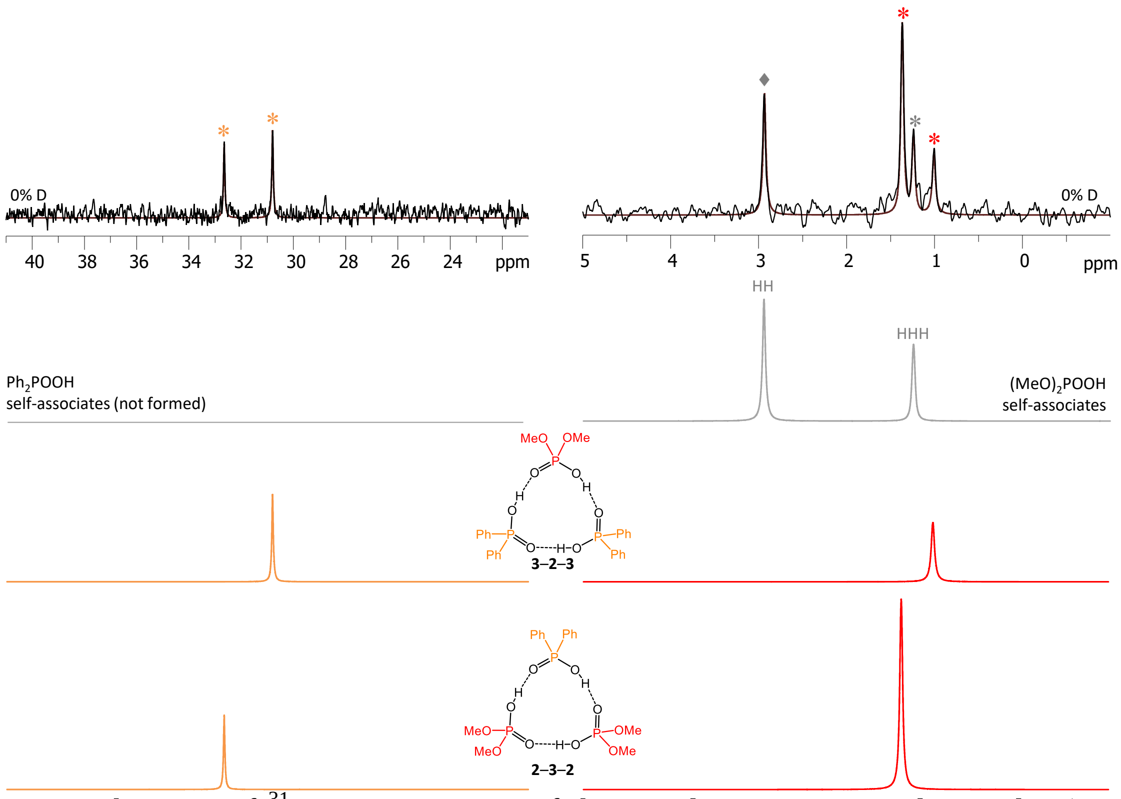

Figure S2. The parts of ^{31}P NMR spectrum of the sample containing acids **2** and **3** (1.8:1) in CDF_3/CDF_2Cl at 100 K. The experimental spectrum is deconvoluted into the sub-spectra arising from self-associated of **2** and two heterotrimers, **2-3-2** and **3-2-3**. The self-associates of **3** are not formed, while the signals of heterodimer **2-3** are not detected due to their low intensity. For visual clarify the signals in the experimental spectrum and the computed sub-spectra are color coded.

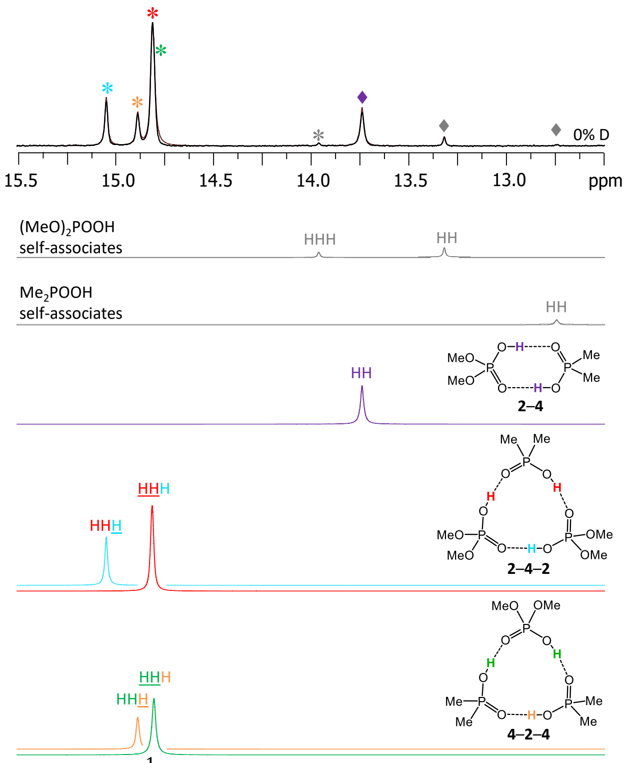

Figure S3. The low-field part of ^{1}H NMR spectrum of the sample containing acids **2** and **4** (1.3:1) in CDF_3/CDF_2Cl at 100 K. The experimental spectrum is deconvoluted into the sub-spectra arising from self-associated of **2** or **4**, heterodimer **2-4** and two heterotrimers, **2-4-2** and **4-2-4**. For visual clarify the signals in the experimental spectrum and the computed sub-spectra are color coded.

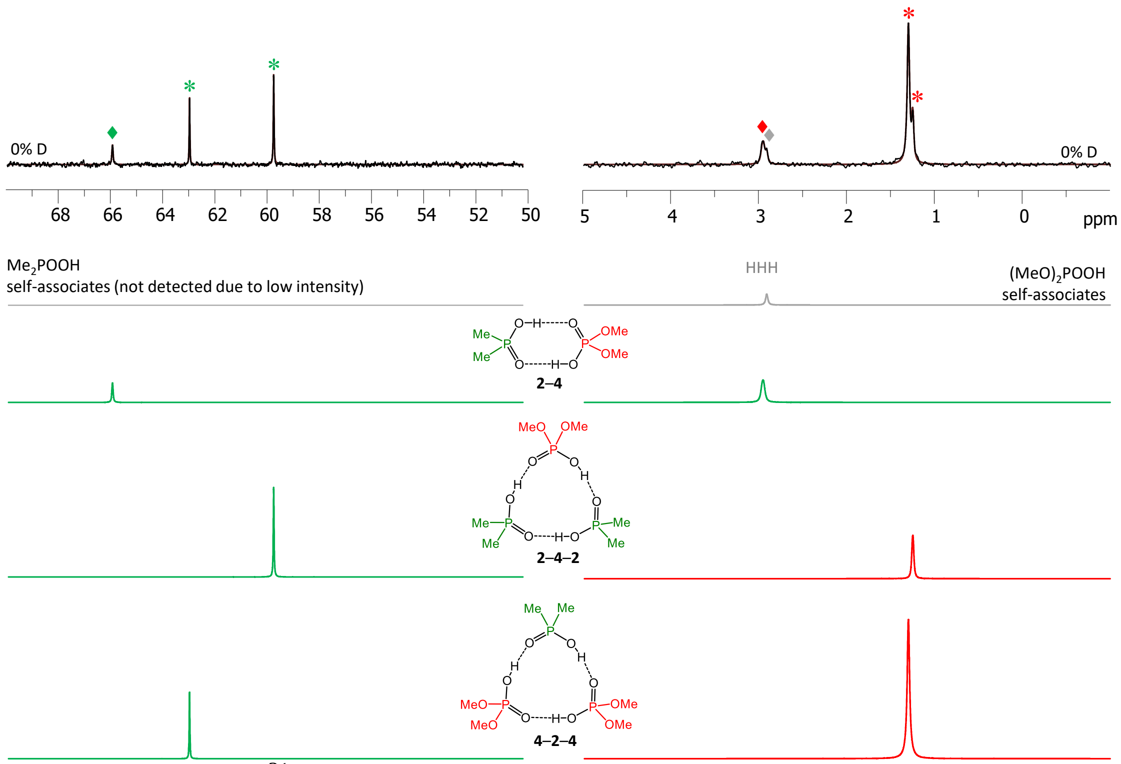

Figure S4. The parts of ^{31}P NMR spectrum of the sample containing acids **2** and **4** (1.3:1) in CDF_3/CDF_2Cl at 100 K. The experimental spectrum is deconvoluted into the sub-spectra arising from self-associated of **2**, heterodimer **2-4** and two heterotrimers, **2-4-2** and **4-2-4**. The self-associates of **4** are not detected due to the low intensity of their signals. For visual clarify the signals in the experimental spectrum and the computed sub-spectra are color coded.

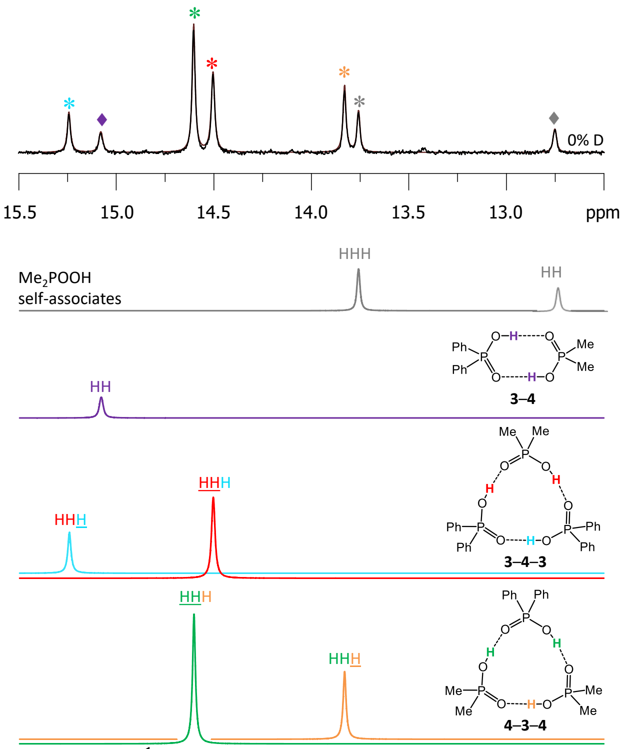

Figure S5. The low-field part of ^{1}H NMR spectrum of the sample containing acids **3** and **4** (1:1.4) in CDF_3/CDF_2Cl at 100 K. The experimental spectrum is deconvoluted into the sub-spectra arising from self-associated of **4**, heterodimer **3-4** and two heterotrimers, **3-4-3** and **4-3-4**. For visual clarify the signals in the experimental spectrum and the computed sub-spectra are color coded.

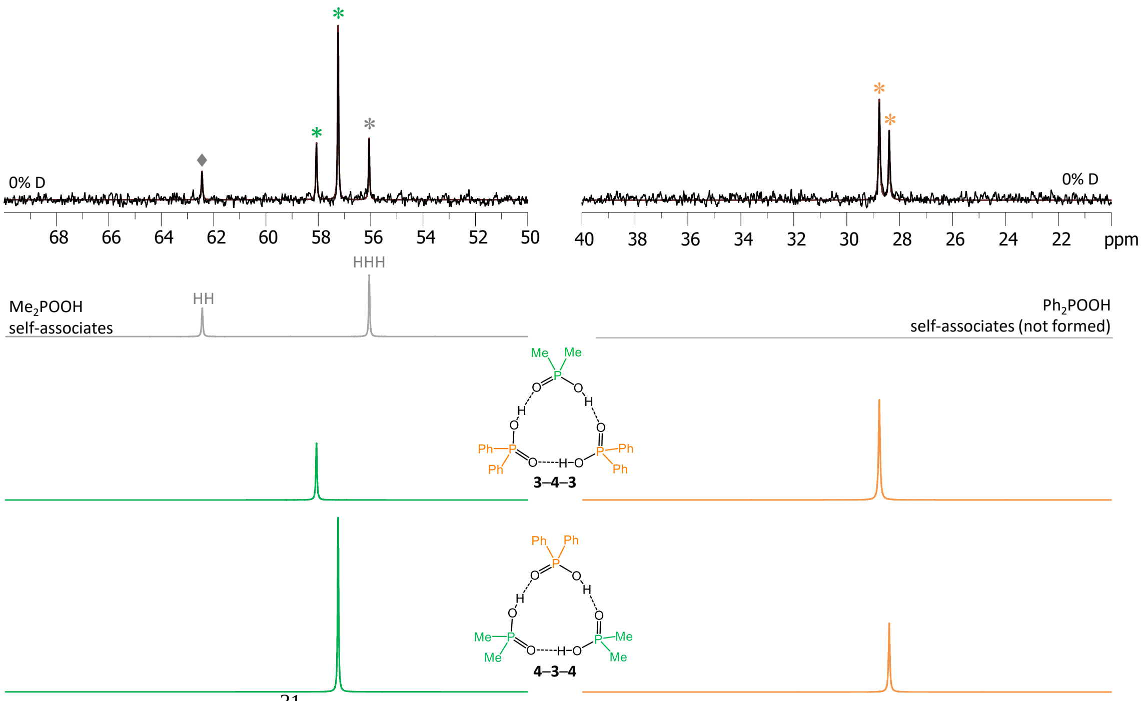

Figure S6. The parts of ^{31}P NMR spectrum of the sample containing acids **3** and **4** (1:1.4) in CDF_3/CDF_2Cl at 100 K. The experimental spectrum is deconvoluted into the sub-spectra arising from self-associated of **4**, heterodimer **3-4** and two heterotrimers, **3-4-3** and **4-3-4**. The self-associates of **3** are not formed, while the signals of heterodimer **3-4** are not detected due to their low intensity. For visual clarify the signals in the experimental spectrum and the computed sub-spectra are color coded.

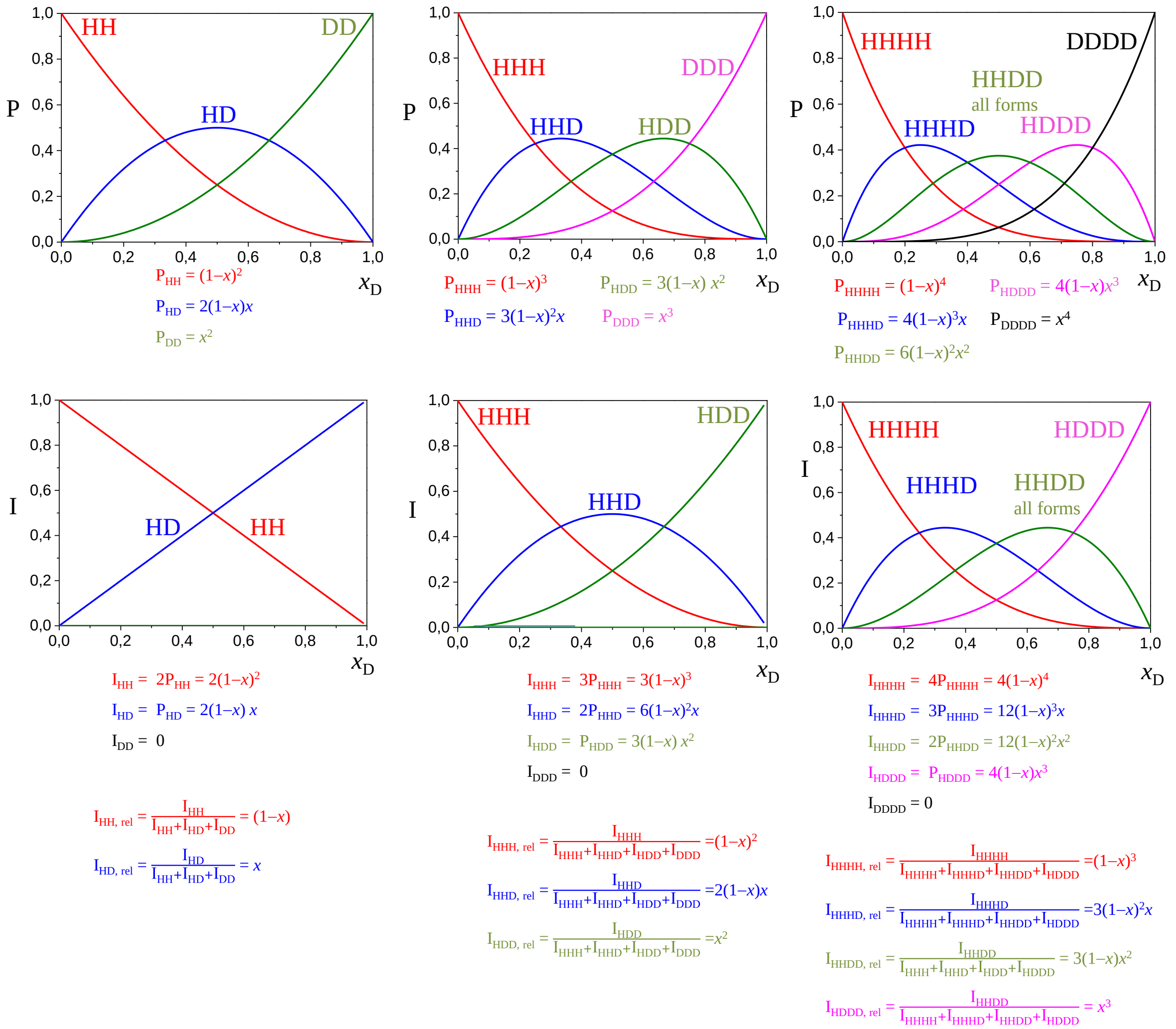

Figure S7. The probabilities of various isotopologs (top) and the relative integrated intensities of their ^{1}H NMR signals (bottom) calculated for cyclic dimers (left) and cyclic trimers (center) and cyclic tetramers of the **X-Y-X-Y** type (right) as a function of the deuteration ratio x_D.

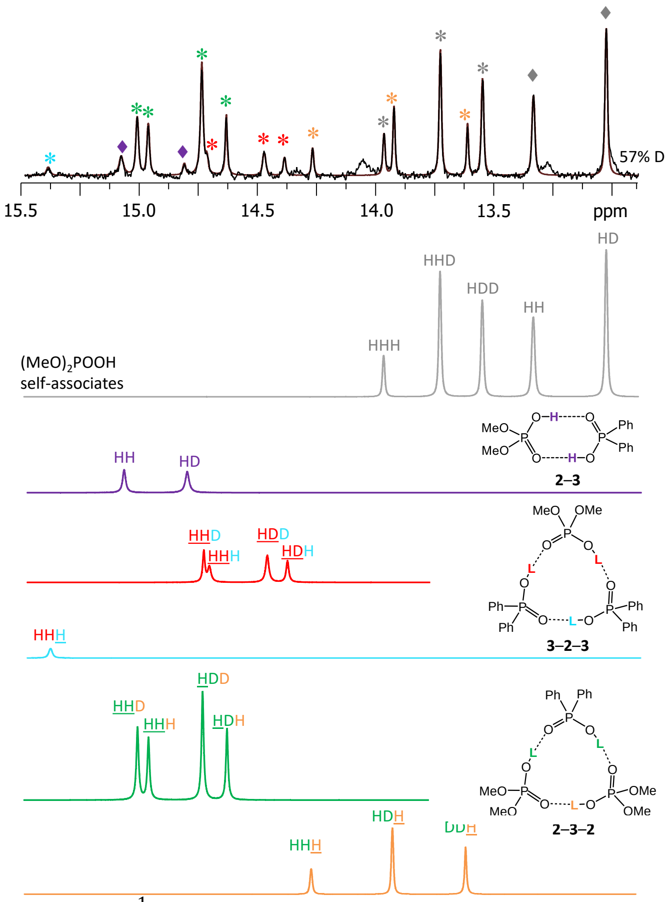

Figure S8. The low-field part of ^{1}H NMR spectrum of the sample containing partially deuterated (OH/OD, 57% D) acids **2** and **3** (4:1) in solution in CDF_3/CDF_2Cl at 100 K. The experimental spectrum is deconvoluted into the sub-spectra arising from various isotopologs of self-associates, heterodimer and heterotrimers. For visual clarify the signals in the experimental spectrum and the computed sub-spectra are color coded.

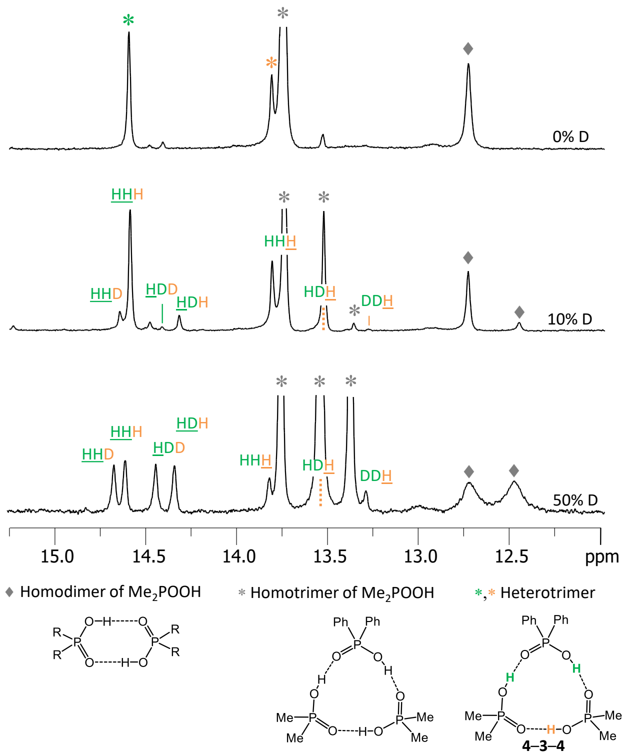

Figure S9. The low-field part of ^{1}H NMR spectra of the sample containing partially deuterated (OH/OD, from top to bottom: 0% D, 10% D, 50% D) acids **3** and **4** (1:22) in solution in CDF_3/CDF_2Cl at 100 K. Due to the large excess of acid **3** only one type of hetero-complex is formed, namely, a heterotrimer **4-3-4**. The evolution of the relative intensities of signals as a function of the deuteration ratio was used to assign the signals to particular isotopologs. For visual clarify the signals in the experimental spectrum and in the computed sub-spectra are color coded.

Table S1. The ^{31}P NMR chemical shifts of various isotopologs of homodimers and heterodimers of POOH-containing acids **1**–**4** in CDF_3/CDF_2Cl at 100 K. The corresponding spectra are shown in Figures 4, 6, 8 and Figures S2, S4, S6.

**Complex**	**$(PhO)_2{}^{31}\underline{P}OOH$**	**$(MeO)_2{}^{31}\underline{P}OOH$**	**$Ph_2{}^{31}\underline{P}OOH$**	**$Me_2{}^{31}\underline{P}OOH$**
**1-1**[a]	–8.09	-	-	-
**2-2**[b]	-	2.91	-	-
**3-3**[c]	-	-	n.d.[d]	-
**4-4**[a]	-	-	-	62.46
**1-2**	–7.88	2.64	-	-
**1-3**	n.m.[e]	-	-	n.m.
**1-4**	–8.17	-	-	72.99
**2-3**	-	n.d.	n.d.	-
**2-4**	-	2.96	-	65.92
**3-4**	-	-	n.d.	n.d.

[a] – chemical shifts found in this work coincide with those reported previously in [Mulloyarova, V.V.; Giba, I.S.; Kostin, M.A.; Denisov, G.S.; Shenderovich, I.G.; Tolstoy, P.M. Cyclic trimers of phosphinic acids in polar aprotic solvent: symmetry, chirality and H/D isotope effects on NMR chemical shifts. *Phys. Chem. Chem. Phys.* **2018**, *20*, 4901–4910.].

[b] – chemical shifts match reasonably well those reported previously in Ref. [Detering, C.; Tolstoy, P.M.; Golubev, N.S.; Denisov, G.S.; Limbach, H.H. Vicinal H/D isotope effects in NMR spectra of complexes with coupled hydrogen bonds: phosphoric acids. *Dokl. Phys. Chem.* **2001**, *379*, 191–193.].

[c] – the diphenylphosphinic acid **3** is poorly soluble in CDF_3/CDF_2Cl and does not form self-associates in a detectable amount

[d] – n.d. – not detected.

[e] – n.m. – not measured.

Table S2. The ^{31}P NMR chemical shifts of various isotopologs of homotrimers and heterotrimers of POOH-containing acids **1**–**4** in CDF_3/CDF_2Cl at 100 K. The corresponding spectra are shown in Figures 4, 6, 8 and Figures S2, S4, S6.

**Complex**	**(PhO)$_2$^{31}POOH**	**(MeO)$_2$^{31}POOH**	**Ph$_2$^{31}POOH**	**Me$_2$^{31}POOH**
**1-1-1**[a]	–11.56	-	-	-
**2-2-2**[b]	-	1.22	-	-
**3-3-3**[c]	-	-	n.d.[d]	-
**4-4-4**[a]	-	-	-	56.07
**1-2-1**	–11.43	1.15	-	-
**2-1-2**	–11.62	1.20	-	-
**1-3-1**	–11.65	-	37.85	-
**3-1-3**	–12.25	-	33.68	-
**1-4-1**	–11.46	-	-	71.55
**4-1-4**	–11.50	-	-	63.86
**2-3-2**	-	1.37	32.65	-
**3-2-3**	-	1.00	30.79	-
**2-4-2**	-	1.30	-	62.93
**4-2-4**	-	1.25	-	59.66
**3-4-3**	-	-	28.76	58.07
**4-3-4**	-	-	28.39	57.24

[a] – chemical shifts found in this work coincide with those reported previously in [Mulloyarova, V.V.; Giba, I.S.; Kostin, M.A.; Denisov, G.S.; Shenderovich, I.G.; Tolstoy, P.M. Cyclic trimers of phosphinic acids in polar aprotic solvent: symmetry, chirality and H/D isotope effects on NMR chemical shifts. *Phys. Chem. Chem. Phys.* **2018**, *20*, 4901–4910.].

[b] – chemical shifts match reasonably well those reported previously in Ref. [Detering, C.; Tolstoy, P.M.; Golubev, N.S.; Denisov, G.S.; Limbach, H.H. Vicinal H/D isotope effects in NMR spectra of complexes with coupled hydrogen bonds: phosphoric acids. *Dokl. Phys. Chem.* **2001**, *379*, 191–193.].

[c] – the diphenylphosphinic acid **3** is poorly soluble in CDF_3/CDF_2Cl and does not form self-associates in a detectable amount

[d] – n.d. – not detected.

$$\underset{complex\text{–}1}{\text{A–H}\cdots\text{B}} + \underset{complex\text{–}2}{\text{X–D}\cdots\text{Y}} \rightleftharpoons \text{A–D}\cdots\text{B} + \text{X–H}\cdots\text{Y}$$

$$fractionation\ factor\ \varphi_{1-2} = \frac{([D]/[H])_{complex-1}}{([D]/[H])_{complex-2}}$$

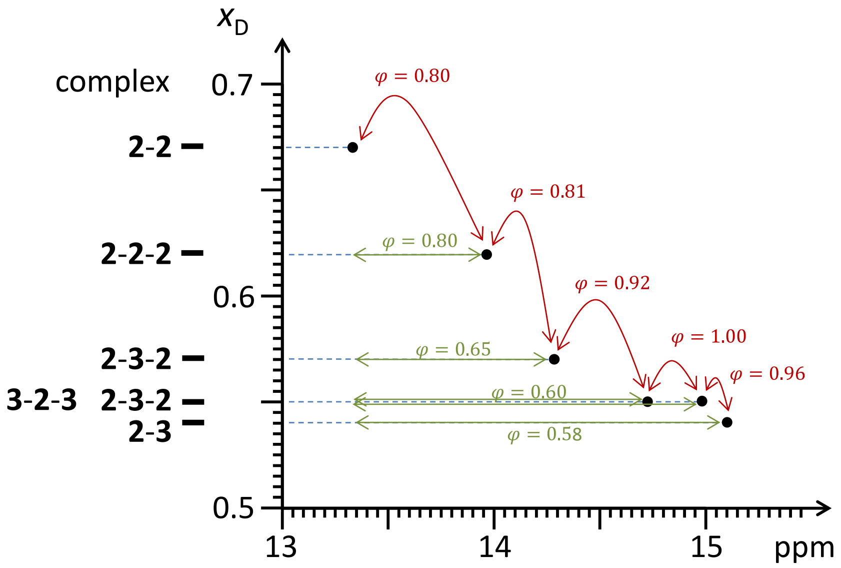

Figure S10. The analysis of H/D fractionation factors for a mixture of partially deuterated acids **2** and **3**, based on the ^{1}H NMR spectrum shown in Figure S8 and the individual deuteration ratios listed in Table S3.

Note: the H/D fractionation factors are defined with respect to a standard/reference, which is usually water. In our case the fractionation factors were calculated either with respect to one of the complexes (green labels in Figure S10) or within a selected pair of complexes (red labels). It has to be mentioned that for many other samples the changes of deuteration ratios x_D between individual complexes are either too small or lie within the experimental error.

Table S3. The deuteration ratios of individual hydrogen bonds for the sample of a mixture of partially deuterated acids **2** and **3** (see ^{1}H NMR spectrum in Figure S8). Note that intensities of some isotopologs are too small to reliably measure the deuteration ratio (this is why only one signal of heterotrimer **3-2-3** was analyzed).

**Complex**	**2-2**	**2-2-2**	**2-3-2**	**3-2-3**	**2-3-2**	**2-3**
**δH, ppm**	13.33	13.96	14.29	14.72	14.98	15.10
x_D	0.67	0.62	0.57	0.55	0.55	0.54
